# Prospects of nutritional interventions in the care of COVID-19 patients

**DOI:** 10.1016/j.heliyon.2021.e06285

**Published:** 2021-02-12

**Authors:** Sabiha Alam, Farhana Rumzum Bhuiyan, Tanvir Hossain Emon, Mahmudul Hasan

**Affiliations:** a Institute of Nutrition and Food Science, University of Dhaka, Bangladesh; b Department of Botany, University of Chittagong, Chittagong, Bangladesh; c Laboratory of Biotechnology and Molecular Biology, Department of Botany, University of Chittagong, Chittagong, Bangladesh; d Department of Genetic Engineering and Biotechnology, Shahjalal University of Science and Technology, Sylhet, Bangladesh; e Department of Pharmaceuticals and Industrial Biotechnology, Sylhet Agricultural University, Sylhet, Bangladesh

**Keywords:** Macronutrients, Micronutrients, COVID-19, SARS-CoV-2, Immunity, Complications, Nutrition, Viral infection

## Abstract

The novel coronavirus disease 2019 (COVID-19) has unfolded an unprecedented worldwide public health emergency with disastrous economic consequences. Around 96 million coronavirus cases have already been identified with over half a million deaths. Despite numerous efforts by the government as well as international organizations, these numbers are still increasing with a surprising rate. Although urgent and absolutely necessary, a reliable therapeutic or vaccine is still elusive and this status quo may remain for an uncertain period of time. Taken that into account, boosting up adaptive immunity through nutritional interventions may help subside this epidemic and save many lives. This review focuses on the nexus between a balanced diet and adaptive immunity, particularly, how a poor diet may lead to compromised immunity resulting in susceptibility to viral infections. Additionally, we discuss how nutrients (vitamins, minerals, trace elements) can be used as a tool to modulate immune response and thus impede viral infections. The study also summarizes nutritional recommendations to combat COVID-19 in different countries and territories as well as dietary sources of those key nutrients. Moreover, different nutritional intervention strategies based on different age groups, physiological and medical conditions were also included, and the challenges of nutritional interventions towards the care of COVID-19 patients are also discussed. Since the availability of a drug or vaccine is still uncertain, a balanced diet or nutrient therapy can be used as a robust strategy to combat COVID-19. Thus, we hope this review may help to make an informed decision with regard to diet choice both at individual level as well as clinical settings.

## 1 Introduction

COVID-19, a deadly respiratory disease caused by a newly emerged coronavirus was first detected in the Wuhan province of China on December 2019 [1]. Within a few days, several patients from Wuhan, China were admitted to the hospitals showing some common symptoms of pneumonia [2]. Now, it has been spread around 215 countries with its pandemic notion [3]. As a consequence of rapid transmission, WHO declared an immediate a public health emergency of international concern (PHEIC) alarm on January 30,2020. Coronavirus is not newly appeared indeed, rather in 2003, the severe acute respiratory syndrome (SARS) outbreak appeared in another state of China (Guangdong, southern China) for 8000 cases and resulted in 800 deaths in 26 countries and characterized as SARS CoV. Later on, in September 2012, MERS-CoV (Middle East respiratory syndrome coronavirus) associated deaths were reported in 858 cases. The disease, novel coronavirus (2019-nCoV) transmission occurred due to SARS-CoV-2 (severe acute respiratory syndrome coronavirus 2) [4, 5].

The basic reproductive number R_0_ of the SARS-CoV-2 in the range of 2.24–3.58 with mean incubation period of 6.4 days had been claimed from the mathematical modeling analysis [6, 7]. The R_0_ higher than 1 implies that the transmission can occur continuously which can cause an epidemic or pandemic if left uncontrolled. Even though the mortality rate is much lower than that of SARS (10.87%) and MERS (34.4%), the highly contagious SARS-CoV-2 confirmed cases around the globe after 6 months of emergence is alarming [8, 9]. Immunocompromised people such as elderly ones, underrepresented minorities and the ones with pre-existing comorbidities are in the high-risk groups of infection. Moreover, like SARS and MERS-CoV, SARS-CoV-2 coronavirus is presumed to escape human immune detection at the initial stage of infection and may dampen the immune function. The vaccine trials for SARS-CoV-2 are going on with some positive results in some developing and developed countries but a certified vaccine is not available yet. In this context, enhancing the body's immune system to combat the disease till launching of an effective vaccine is necessary. However, in an organism, the immune system is a key performer that compromises defense not only for the common diseases and health complications like abnormal cell development and cancers, arthritis, allergies, but also from pathogenic infections by bacteria [10] and viruses including novel coronavirus (COVID-19). The immune defense system uses numerous plasma proteins (blood proteins, Immunoglobulin G (IgG), hemopexin (heme-binding protein) to modulate the immune response [11]. Besides, great nourishment is fundamental to build a strong immune system whereas malnutrition, a global problem, is considered as the most predominant cause of immunodeficiency worldwide [12, 13]. In that case, a balanced diet can ensure proper nutrition (carbohydrate, protein, fat, fiber, vitamins, and minerals) which is essential to strong immunity [14]. Diet is a particular selection of food and drink which is regularly consumed by a person to improve one's physical condition to prevent or treat a disease, and thus assist to keep an individual mentally and physically healthy whereas a balanced diet includes distinct food groups in certain quantities and proportions to fulfill the requirement for calories, proteins, minerals and vitamins. There is no alternative to a balanced diet to keep ourselves physically and mentally fit.

**Figure 1:**
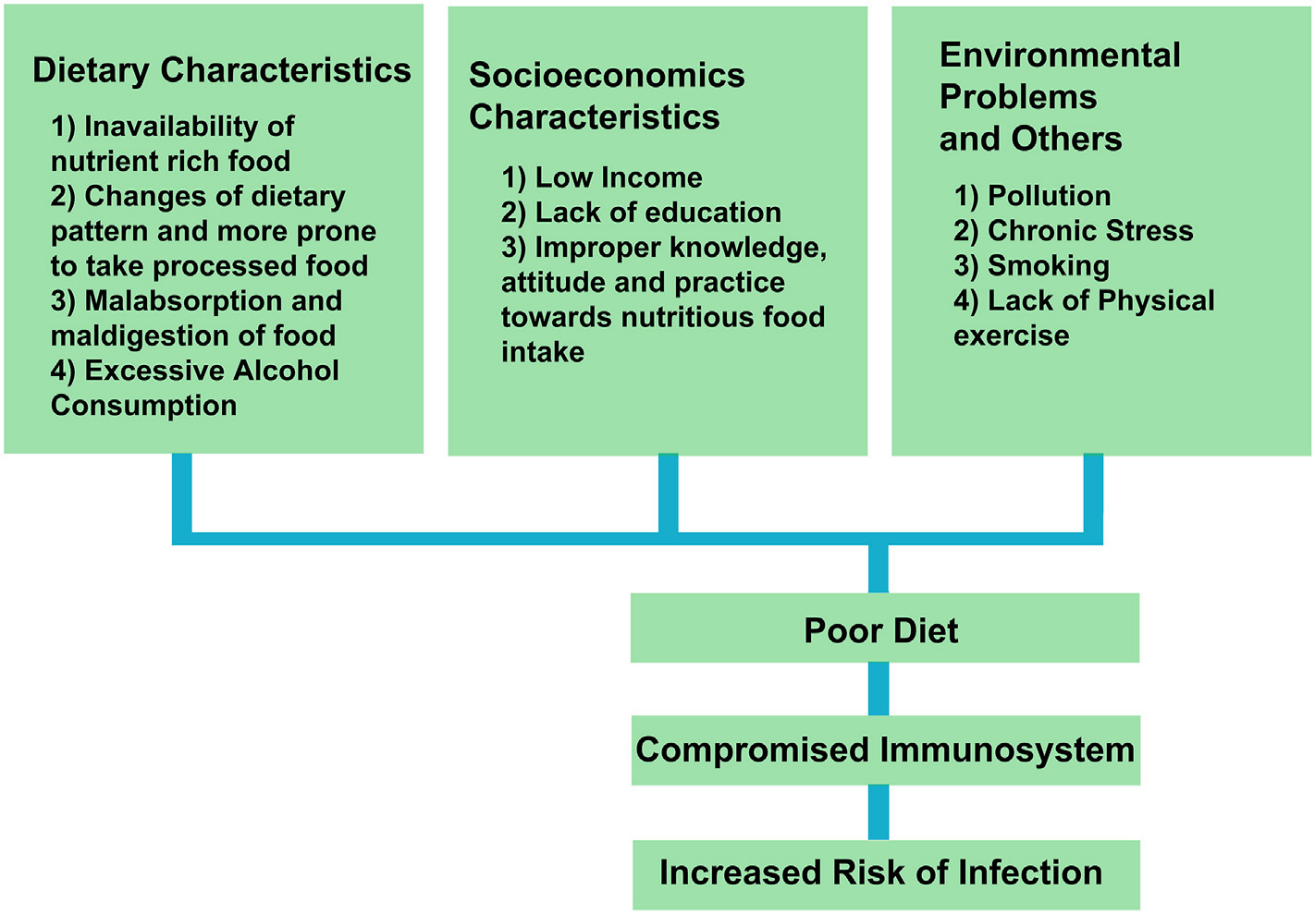
Leading factors towards the higher risk of infection.

Immunity refers to the capability of the organism to fight against the attack of microbes and harmful substances [15]. Lack of a balanced diet, poor socioeconomic conditions, health complications, irregularity in physical activities, environmental pollution altogether lead to poor diet followed by compromised immune systems (Figure 1) which ultimately results in an increased risk of infection by pathogens [16]. Hence, taking a healthy diet, ensuring proper nutrition, and maintaining social distance can be the best way as preventive methods to overcome the battle against the SARS CoV-2. Several researchers are focusing more on the modification of diet to treat the deadly COVID-19 worldwide. Nutritional interventions can work well in this regard to save people from unexpected health complications and deaths. Therefore, this review attempts to know about the potential role of nutrients of different food groups with their antiviral properties to increase immunity against viral infections including SARS CoV-1 and other RNA viruses by enabling people to make a right diet choice in pandemic as well as post pandemic situation.

## 2 Methodology

Related peer reviewed scientific articles, letters, case study or other documents are screened from different journal depositories (e.g., Google Scholar, PubMed, NCBI) by searching specific keywords related to COVID-19 and nutritional interventions. About 162 articles or scientific documents are studied for scrutinizing the data to develop the review.

### 2.1 Global needs of nutritional interventions towards the COVID-19 pandemic

The COVID-19 infection is spreading alarmingly infecting a huge number of people after its first detection in Wuhan. Data retrieved from 32 isolated places illustrated that the median infection fatality rate was 0.27% till July, 2020 [17]. Although the viral transmission was supposed to be connected with the trade animals in the market, there is no conclusive evidence of animal association to date with the COVID-19 infection [18]. Respiratory droplets or close attachment with the affected ones were identified as the core medium of human-to-human spreading of this virus, and it was also confirmed by different studies [19, 20]. Asymptomatic viral shedding has caused more severity of this contagious disease leading to high risk of infection [20]. The elderly people are more prone to infection compared to the young and young adult ones due to shrinkage of naïve T cells for prolonged antigen encounters [21]. Moreover, the people having comorbidities like diabetes, hypertension and other cardiovascular and cerebrovascular diseases are more likely to be affected in a severe way than healthy population [22, 23, 24, 25].

SARS-CoV-2 virus can be latent for about 2–14 days in the host leading to non-severe symptomatic infection to acute respiratory distress syndrome (ARDS) with increased viral load [26, 27]. In addition, the SARS CoV-2 pathogenesis is accompanied by the response of inflammatory system due to cytokine storm and causes an excessive inflammatory reaction occurred by immune response of host individuals after the induction of SARS-CoV-2 infection. During infection, SARS CoV-2 usually causes human body attachment through different organs like mouth, nose or eyes and enters into the cell through fusion using the cellular transmembrane proteins serine protease 2 (TMPRSS2), furin, along with viral receptor ACE2 (Angiotensin-converting enzyme 2). Then, genetic material of the virion particle is transferred to the host cell cytoplasm after removing envelope and capsid part of it, and this allows the translation of viral polypeptides. Later, 16 essential non-structural proteins (NSPs) are generated after the slicing of polypeptides by chymotrypsin-like protease (3CLpro), and they facilitate further cellular events such as replication, transcription etc. [28]. Then, infected cells produce a large volume of cytokine molecules when they become compromised by SARS CoV-2. The assembly of SARS CoV-2 into the lumen of the ERGIC (Endoplasmic Reticulum Golgi Intermediate Compartment) is supported by the immune system supports for the new copies of virion particles followed by leaving the cells through exocytosis [29]. Increased level of cytokines and chemokines have been identified in SARS CoV-2 infected patients which can lead a patient to a critical stage [30, 31]. Specially, Type I IFN (IFN–I) response and its downstream cascade play a crucial role in counteracting the viral replication by inducing the adaptive immune system to produce antibody and memory cells. SARS-CoV viruses block the expression of antiviral cytokines IFN-αβ and up-regulate the pro-inflammatory cytokines and chemokines. The uncontrolled production of cytokines leads to the dysregulation of the immune system resulting in cytokine storm and induces apoptosis in different organ tissues and eventually death [32, 33]. Severe hypercytokinemia or cytokine storm cases were reported in China, Italy, USA, Spain, Brazil and many other countries. As there is no effective treatment available for the disease, boosting immune response in the preliminary asymptomatic stage is crucial to maintain a good health. Long time and consistent healthy dietary pattern is the key determinant of sound health. On the contrary, unhealthy diet and lifestyle promote the development of non-communicable diseases like diabetes, cardiovascular disease and chronic respiratory disease which have negative impact on prognosis of COVID-19 [34]. An ideal nutritional condition is the prerequisite for regulating the oxidative stress and inflammatory process which ultimately have impact on immune system [35]. Nutrition deficiency resulted into different complications act as negative prognosis factor in COVID-19 patients. Host nutritional status can predict the susceptibility of individuals to be infected with SARS CoV-2. The diabetic and cardiovascular patients have increased number of ACE2 receptor in adipose tissue due to treatment with ACE inhibitors as well as angiotensin II type 1 receptor blocker drugs and are more susceptible to COVID-19 [36]. Malnutrition, more specifically under-nutrition as well as over nutrition may promote viral replication through altering the immune system in cells. People with obesity, diabetes, cancer, cardiovascular diseases are the most severely affected individuals with SARS CoV-2. Basically, immune cells require high energy to perform their regular functions but when cells are infected, their nutritional requirement increases dramatically for the activation of immune response. Malnutrition addresses a significant reduction of immune cells, e.g., CD4+, CD8+ T cell numbers [37]. Besides, during both under-nutrition and over-nutrition period, the modulating factors like hormones and cytokines induce the reduction of nutrient consumption, thus our body is more defenseless against infection. Among obese people, when specific inflammatory cytokines become preactivated and expanded in adipose tissue, it results in reduced antigen response and impedes the regular functions of natural killer cells, dendritic cells and macrophages [38, 39]. ROS reacting with DNA induce the modification of DNA strand bases and DNA protein cross-links which leads to carcinogenesis in the human body [40].

Low pre-albumin level in circulating blood marks malnutrition is a potential predictor for the prognosis of COVID patients to the acute respiratory problems leading to the ventilation [41]. Lymphocytopenia, a malnutrition marker, has been observed mostly in non-survivor patients than the survivor ones. Obesity caused by consumption of food high in saturated fat, sugar and carbohydrate has link to high mortality and increases risk of influenza-related complications. Obese and obese like diabetic patient exhibit weaker immune response during antigen presentation due to reduced macrophage activation and cause increased susceptibility to viral infection [42]. Therefore, the nutritional or dietary interventions can be an efficient strategy to boost up the immunity of people who can be able to combat further against COVID-19 infection [43].

### 2.2 Viral susceptibility and common complications associated with nutrient deficiencies

**Figure 2:**
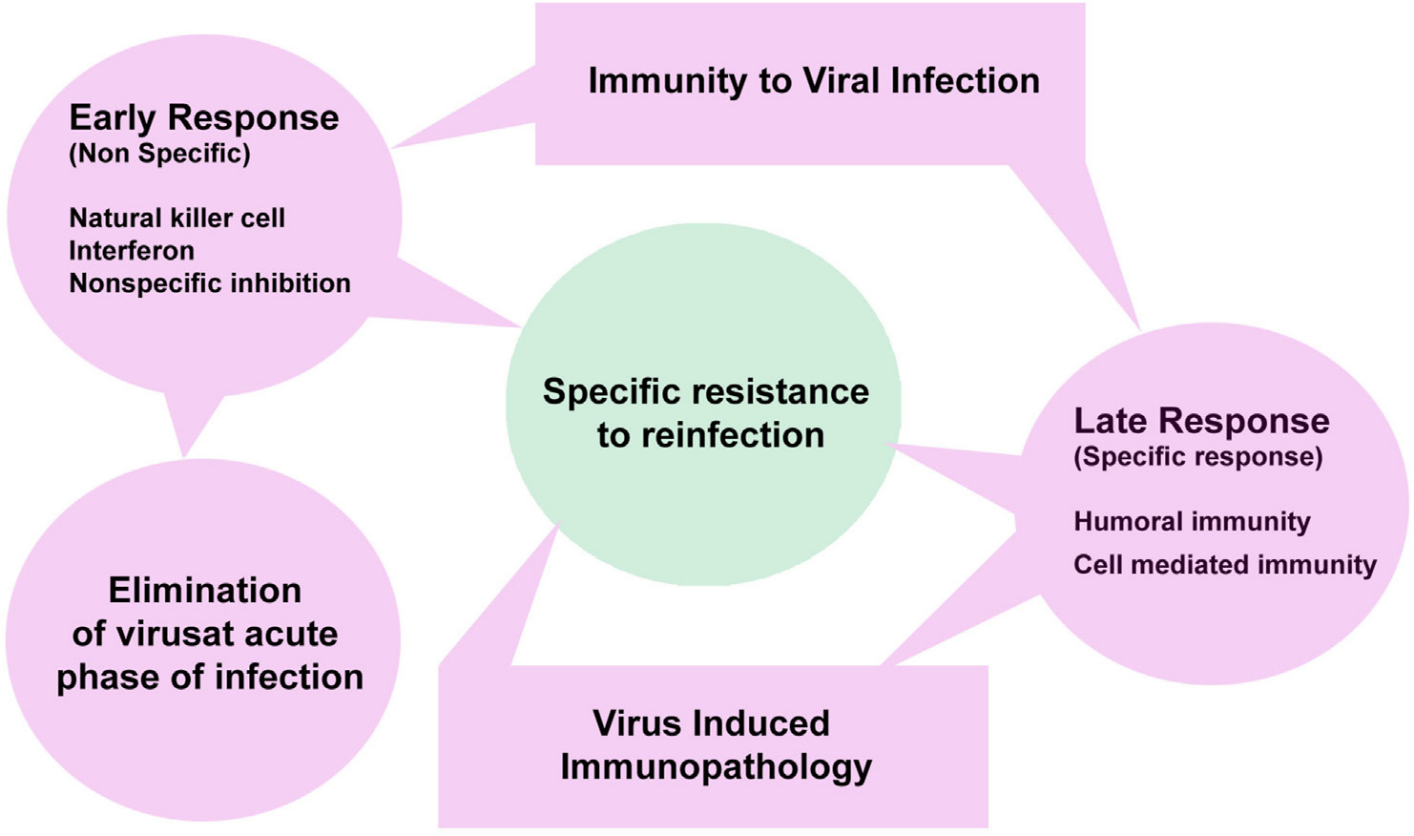
Associated immune response of a virus infected person.

The immune system comprises two lines of defense including adaptive immunity and innate immunity [44]. Innate immunity is the rapid immunological, non-specific mechanism to protect the host from an invading pathogen [45] whereas adaptive immunity is the antigen-specific mechanism against virus infection [46]. Basically, the virus interaction with the host and spreading strategy of the virus decide the immune response of a patient [47]. On the basis of infection stage in the patient and spreading route, antigenic existence of viral molecules could be detected in various parts of the body. Besides, the host has diverse immune defense functions (humoral immunity through IgA and cell-mediated immunity can eliminate local viral infections). Humoral immunity stimulates B lymphocytes to produce viral antigen-specific antibodies [48, 49]. Virus recognition by leukocytes of virus-infected cells, cytokines (growth factors that are secreted by certain cells of the immune system) production is stimulated by the virus-infected cells or the virus [50]. However, virus-infected cells could be identified and killed by natural killer (NK) cells, cytotoxic T lymphocytes or macrophages. Helper T cells can also identify virus-infected cells and generates numerous essential cytokines [51, 52]. Monocytes (monokines), T cells, and natural killer cells (lymphocytes) usually cause the production of cytokines which contributes a significant role to regulate immune functions and develop antiviral immune functions [53] (Figure 2). However, the nutritional status of a person has an impact on immune cell metabolism and function [54].

Besides, sufficient intake of carbohydrate aids to maintain stable blood sugar level and reduces body's stress response by modifying the undesirable mobilization of immune cells [55]. Moreover, the severity of protein deficiency affects the mechanisms of primary lymphoid organs (bone marrow, thymus) leading them to generate B and T cell repertoires and reduces the generation of IL-6 and TNF-Alpha by bone marrow cells [56, 57]. Vitamins and minerals help optimizing the innate immunity through development, differentiation and chemotaxis of innate cells; activating macrophage and neutrophils killing property and producing antimicrobial proteins. These nutrients also have pleiotropic effect on adaptive immunity and foster the immune function via antibody production and memory cell generation [58]. Deficiency of nutrients, whether they are macronutrients (carbohydrates, protein, fat) or micronutrients (vitamins, minerals, trace elements) can lead to impaired immune systems [59] and can increase the risk of bacterial and viral attack [60, 61, 62]. Moreover, inadequate macronutrients or specific micronutrients, especially vitamins and the minerals magnesium, zinc, iron, selenium can cause clinically significant immune deficiency and infections in humans [63].

For example, vitamin C deficiency had been reported for susceptibility to respiratory infections like Pneumonia [64]. Similarly, low level of 25-hydroxyvitamin D, a major vitamin D metabolite have been found associated with acute respiratory tract infection [65]. On the contrary, different micronutrients can be achieved from our daily food which can help reduce inflammation, improve immunity due to their antiviral activities [66, 67, 68]. Vitamins and minerals help optimizing innate immunity by production, differentiation and chemotaxis of innate immune cells and activating macrophage. Common health complications associated with nutrient deficiencies were enlisted in the Table 1 [69, 70, 71, 72, 73, 74, 75, 76, 77, 78, 79, 80, 81, 82, 83, 84, 85].

**Table 1:** Common health issues and immunity involved health complications associated with nutrient deficiencies.

Nutrients deficiency	Consequences (Immunity involved health issues)	Ref.
Vitamin A (antioxidant)	I) Impaired of innate immunity (frequent throat and chest infections), hematopoiesis and typical ocular effects II) Stunted growth in children III) Increased infertility and trouble conceiving among women	[59]
		[60]
Vitamin B1	I)Shortness of breath II)Reduced reflexes and muscle weakness	[61]
Vitamin B6 (cofactor of hemoglobin synthesis)	I) Hampered the hemoglobin synthesis that leads to decrease in oxygen level in the human body	[62]
Vitamin B2	I)Normochromic-normocytic anemia II)Increased oxidative stress, inflammation and cell proliferation	[63]
Vitamin C (Antioxidant)	I)Poor immunity II) Pneumonia III) Impaired bone growth in children	[64]
Vitamin D	I) Increased replication rate of viruses II)Declined concentration of pro-inflammatory substances (cytokines)	[65]
Vitamin E (Antioxidant)	I)Increased inflammation rate II) Retard physical and mental growth in children	[66]
		[67]
Magnesium (Electrolyte)	II) Impaired innate and acquired immune responses as well as immunoglobulin synthesis	[68, 69]
Iron	II) Impaired immune cells proliferation and maturation and a particular response to infection II) Cognitive and behavioral problems in children III)Recurrent acute respiratory tract infections	[70, 71, 72]
Zinc (Antioxidant)	I) Impaired the outgrowth and activation of T lymphocytes, B lymphocytes, antibody production (specifically Immunoglobulin G) and decreased eosinophils concentration, II) Retarded fetus growth by causing the recurrent abortion in pregnant women	[73, 74]
Selenium	I) Induced the mutation of innocuous strains of RNA viruses (Influenza virus, Coxsackie viruses) to heavily pathogenic strains II) Accountable for recurrent abortion in pregnant women	[75]

### 2.3 Food nutrients with antiviral activities suggested for COVID-19

Coronavirus is considered the worst ever health disaster in the recent eras. Nutrients being classified into two major groups such as macronutrients and micronutrients, play a cornerstone role in human body. Macronutrients considered as the energy-providing nutrients like carbohydrates, protein, fat and dietary fiber are needed to be consumed in larger quantities (g) as they regulate regular life processes (growth, development, repair of tissues) as well as maintain body functions to carry out daily life activities. Besides, diet habit is not same around the world and diet is linked to immune function [86]. Therefore, suppressive immune systems cases have been increasing recently all over the world especially middle and low-income countries. However, in immune system, the dietary factors that cause malfunction are the insufficient intakes of energy and macronutrients [87].

Micronutrients are minerals and vitamins which are required in very tiny amounts [88, 89]. Albeit, together both are extremely important for the normal body functioning in human body [90, 91]. They are involved in triggering many important biochemical reactions for example-works as cofactors and coenzymes in metabolism [92, 93, 94, 95]. However, micronutrients are getting large attention all over the world during the COVID-19 pandemic for its ability to alter the susceptibility to infection [96, 97, 98]. Immunity involves vitamins renovating the capability of some cells to produce certain cytokines that affect the mechanism of immune cells [99]. Vitamin E is indispensable to get rid of chronic viral infections [100]. Different water-soluble vitamins like vitamin B complexes, vitamin C, and fat-soluble vitamins (Vitamin A, vitamin D, vitamin E), different trace elements (Zinc, Magnesium, Iron, Selenium) have been proved to show the satisfactory effect on enhancing human immune response. Adequacy of iron can protect from the respiratory tract infections in severely critically infected coronavirus patients.

Fatty acids are structural components of cell membranes. They are known as energy source, signaling molecules, precursors for the synthesis of eicosanoids and similar mediators and are linked to play various roles in immune cells [101, 102, 103]. Essential fatty acids such as omega-3 fatty acids modulate immune function by its action on inflammatory response [104]. The source of essential fatty acids can be found in the Table 2. Magnesium is associated with the immune system in both innate and acquired responses. It acts as a cofactor for participation in immunoglobulin synthesis and antibody production. Magnesium is the most overlooked electrolyte, although it has an enormous role in immune function [105]. Along with these, antioxidant naturally found in plant-based food have profound impact as antiviral agent. SARS-CoV-2 infected cells face imbalance between oxygen reactive molecules and free radicals which creates oxidative stress resulted in pro-inflammation [106]. Hence addressing antioxidant nutrients in our diet can reverse the inflammatory reaction induced by cytokines in human body [107]. Antioxidants can inhibit oxidation reaction and thus prevent the production of free radicals in cytoplasm that may damage the cells through chain reactions [108]. Metabolic disorders and respiratory infections, more specifically lung damages resulting from cytokine production may be altered by introducing antioxidants in our meals [109, 110]. Different phenolic compounds that are considered as the secondary plant metabolites function as the natural antioxidants. Starchy food like potato, yam; dark green leafy vegetables like kale, Brussels sprouts, broccoli as well as cereals, legumes, in addition to species such as parsley, rosemary, quercetin along with fruits-red grapes, blueberries, cranberries and animal-based food milk contain a rich amount of phenols [111]. Apart from the dietary sources, medicinal plants are considered as one of the significant sources of antioxidants [112]. Hence, the good nutritional status of the host contributes a major role to deal with different infectious diseases [113]. Therefore, proper nutrition must be ensured to deal with the unexpected infections of the patients who are vulnerable or who have already been attacked by the novel coronavirus. If through diet, the daily required amount of different nutrients is not met, different processed food, and fortified with different nutrients can be approached to ensure a healthy lifestyle. Literature studies found a significant role of some food nutrients to tackle a few harmful RNA viruses including SARS viruses through boosting up immunity, and these were presented meticulously in the Table 2 [114, 115, 116, 117, 118, 119, 120, 121, 122].

**Table 2:** General supportive and key food items to stimulate human immune system.

Nutrition Interventions	Susceptible viruses	Major Food groups			Ref.
		Plant origin	Animal origin	Others (Processed food and nutrient supplementation)	
Fat-soluble Vitamin A	Measles virus, Human Immunodeficiency Virus (HIV), Avian Coronavirus	Orange and yellow vegetables, fruits,broccoli, most dark green vegetables, spinach	Eggs, cod liver oil, beef liver	Fortified skim milk	[92]
Water-soluble Vitamin B complexes	MERS-CoV; Ventilator-induced Lung Injury	Brown rice, legumes, sunflower seeds and nuts, fruits (bananas, citrus fruits), dark leafy vegetables	Red meat, poultry, fish, eggs, salmon, liver and other organ meats, milk, cheese, oysters, mussels, pork	Cheese, yogurt, nutritional and brewer's yeasts, fortified cereal	[93]
Vitamin B6	HIV	Bread, whole grain cereals (brown rice, oat meal), vegetables, Soybean, potatoes, banana, spinach, seeds, carrot	Pork, fish, poultry (Chicken, turkey), eggs, milk, beef liver, beef	Vitamin B6 can be used as dietary supplement	[94]
Vitamin C	Avian Coronavirus; Lower respiratory tract infections	Citrus fruits, broccoli, cauliflower, sweet potato, strawberries, tomatoes, papaya	Beef liver, oysters, pork liver, eggs	Vitamin C tablets can be taken as a supplementation	[95]
Vitamin D	Bovine Coronavirus	seaweeds, oat, soy milk, cereal,	Marine fish, beef liver, cheese, egg yolk, milk, shrimp, mushrooms	Cheese, fortified soy milk, fortified cereal, Vitamin D tablets can be taken as a supplementation	[96]
Vitamin E	Coxsackie Virus, Bovine Coronavirus, HIV virus	Vegetable oils, nuts, seeds, green leafy vegetables	Marine fish, octopus, goose meat	Vitamin E fortified oil, Vitamin E capsule can be taken as a supplement	[97, 98, 99]
Omega-3 polyunsaturated fatty acids (PUFA)	Influenza virus, Human Immunodeficiency virus	walnuts, canola oil, spinach, soybeans	Marine fish, shrimp, oysters,	Omega-3 fatty acids can be used as supplements	[100]
Magnesium		Green leafy vegetables, fruits (banana, avocado), nuts, seeds, legumes, peas, spinach, oatmeal	seafood (Salmon, mackerel, tuna), shrimp, egg, milk, beef, chicken,	Magnesium pills	[101]
Iron	Viral mutations	Legumes, pumpkin seeds, nuts, oats, brown rice, spinach, beans, potatoes	organ meats, beef spleen, pork liver, clams, egg yolk, shrimp	Dark chocolate, meanwhile Iron the tablet can be taken as a supplementation	[102]
Zinc	Measles virus, SARS-CoV	nuts, sesame seeds, pumpkin seeds, soybeans whole grains	Meat, shellfish, dairy products, eggs, poultry	Cheese, dark chocolate, cocoa powder	[52]
Selenium	Influenza Virus, Avian Coronavirus; Viral mutations	Almonds, pumpkin seeds, sunflower seeds, whole wheat bread	Fish, eggs pork, beef, chicken, turkey		[103]
Tannins	Influenza Virus	Tea (Green Tea, Oolong Tea, Black Tea, Puer Tea), berries, walnuts	Not available in animals	wine, chocolates	[104]
Essential oils	reducing flu virus (RNA virus) activity in vitro	carrot seed, cinnamon bark, clove bud, sweet orange, eucalyptus, rosemary, and orange, lemon	Not available in animals	Essential oils are available as supplements	[105, 106]

### 2.4 Nutritional intervention strategies during COVID-19 pandemic

Nutritional intervention is a planned action that can be implemented to bring out a beneficial alteration in nutrition-related behavior; health condition for a person, a target group of people playing a key role to combat deadly diseases like coronavirus disease [123]. During the coronavirus pandemic outbreak in China, initially, the aged individuals were infected mostly. Although at the very beginning of the COVID-19 outbreak, there was a lower rate of infection among infants and neonates, gradually, through mutation of the virus, it has been changing the genetic material, attacking infants and causing human deaths over time. Development of immune responses varies among different age groups along with gender, physiological conditions and activities [124]. The older malnourished adults are more likely to have inferior health outcomes, longer hospital stays, and increased mortality rate. Therefore, effective defensive strategies to promote good nutrition among older populations are desired [125, 126]. A study showed that different factors for instance: aging, immunity, virus infection fatality rate are strongly interlinked in human body [127]. The innate, immature, and adaptive immune system, which matures and acquires memory, goes into a decline in adulthood followed by the risks of various kinds of infection [125]. However, vitamin C, vitamin A, vitamin D, Zinc, Iron, Magnesium, vitamin b-12 are being used to treat coronavirus patients worldwide with a hope of saving million lives. Literature searches found the application of nutritional interventions (mostly of vitamin C and vitamin D) in few countries like China, Italy, the USA and Iran, and some other countries. They are also taking into account the application of those nutrients on coronavirus patients seriously (Table 3) [128, 129, 130, 131, 132, 133, 134, 135, 136].

**Table 3:** Current status of nutritional intervention strategies for COVID-19 patients.

Nutritional Intervention	Nutrient Type	Applied Nutrients	Mode of Actions against COVID-19	Referred Countries	Ref.
Dietary approach and supplementation	Micronutrient	Vitamin C	1)Inhibit cytokine storm through reducing inflammation rate 2) Reduce respiratory tract infection	China, Italy, USA, Iran, Bangladesh	[112] [113]
Dietary approach and supplementation	Micronutrient	Vitamin D	1)Vitamin D tablets can be taken to reduce mortality rate 2)Suppress cytokine storm in human body	China, France, Italy, USA, Germany, Iran, South Korea, Philippine, Indonesia	[114] [115] [116]
Dietary approach and supplementation	Micronutrient	Zinc	Hypothesized to treat COVID-19 patients with it due to its antiviral activities and modulation of immune response	University of Melbourn proposed for the world first trial	[117]
Combined supplementation	Micronutrients	Magnesium, Vitamin B12, Vitamin D	Reduce patients' demands for oxygen support and intensive care support	China	[118]
Oral Supplementation	Macronutrient (Protein)	High dose oral and/or IV Glutathione	Reduces respiratory symptoms	New York, USA	[119]
Food supplementation	Micronutrients	Copper, Iodine, Selenium, Zinc	Immune enhancers towards SARS CoV 2	Egypt	[120]

However, there are three types of nutritional intervention which are proposed worldwide to resolve nutrition problems: 1) dietary approaches, 2) fortified and 3) supplementary. Modification of diet might be one of the best approaches. Due to the safety, cost-effectiveness and efficiency for assisting human immune system to combat against COVID-19, dietary supplementation is getting worldwide attention. In a recent RCT study in USA, a decrease in mortality rate was observed when 167 patients with sepsis-related ARAS were given 15 mg/day IV vitamin C [137]. In a multi-nominal logistic regression model, a retrospective study conducted on 212 people in Philippine showed a significant association of serum vitamin D status with COVID-19 patients' clinical outcomes [138]. People who are more prone to Influenza or COVID-19, should take 10,000 IU/d vitamin D3 doses for a several weeks to increase serum 25(OH) D concentrations, followed by 5000 IU/d [139]. Vitamin E, being an antioxidant, it can reduce the rate of inflammation [140]. Optimum level of vitamin E is indispensable to get rid of chronic viral infections [141]. Therefore, vitamin E must be taken in an adequate portion on a regular basis to reduce the possibility of being infected by SARS CoV-2. But, unfortunately, vitamin E got little attention from medical practitioners as a potential nutritional therapy for COVID-19. Vitamin B-complexes also have enormous role to treat COVID-19. Neutrophil infiltration into the lungs could be significantly inhibited by vitamin B3 treatment which also has anti-inflammatory effect during ventilator induced lung injury. However, Blood coagulation is observed in COVID-19 patients leading to deaths [142]. Iron rich food along with vitamin B6 rich food are equally important. Around 70% of ‘Iron’ is found in hemoglobin, which carries oxygen to different cells in human body [143]. Vitamin B6 can introduce a new insight to treat COVID-19 patients. Perhaps, among COVID-19 patients, due to their lower oxygen level, they sometimes face critical phrase [144]. In that case, to level up their oxygen level, consuming functions as a cofactor in hemoglobin synthesis [145]. If our body faces vitamin B6 deficiency, it will directly hamper the hemoglobin synthesis leading to decrease oxygen level in human body. In severe cases, it is one of the main reasons behind million deaths. Vitamin B6 rich could be an alternative solution in that case. In contrast, deficiency of selenium can be the cause of the mutation of innocuous strains of RNA viruses (Influenza virus, Coxsackie viruses) to heavily pathogenic strains [146]. But there is no credible study done on Selenium to ensure its impact on SARS-CoV-2. Zinc deficiency associated with cardiovascular dysfunction, obesity, diabetes, cancer and age-related complications may be considered as a useful treatment due to its antiviral activity and regulation of inflammatory response [147]. Albeit, till to date, no randomized control trial has been done to depict the real impact of Zinc on coronavirus patients. However, SARS CoV-2 interferes heme metabolism in human body through attacking 1-beta chain of hemoglobin and finally capturing porphyrin resulting in Iron deficiency [148]. Deficiency of iron has been acknowledged as a remarkable reason behind the development of recurrent acute respiratory tract infections [149]. As a matter of fact, adequacy of iron can contribute a leading role in the improvement of respiratory tract infections in severely critical infected coronavirus patients. In china, a cohort study of old age people showed positive feedback as most of the patients' demands for oxygen support or intensive care support were reduced [150]. Still, a randomized control trial including a large population is needed to observe the true benefits of those nutrients' combination on COVID-19 patients. Supplementation can be applied for adults if their dietary components cannot meet the Recommended Dietary Allowance (Table 4) [151, 152, 153, 154, 155, 156, 157, 158, 159, 160, 161, 162, 163, 164, 165, 166, 167, 168, 169, 170, 171, 172, 173, 174, 175].

**Table 4:** Nutrient recommendations of COVID-19 patients based on different age groups and physiological conditions.

Target groups	Complications	Recommendations	Health Benefits	Ref.
Pregnant and lactating women	Iron, zinc, calcium, vitamin A, vitamin D, and folic acid deficiency	Protein, Zn, Ca, and folate-rich food. Supplementation is prohibited for infants.	Reduces infection in child by increasing the immune response	[129, 130] [131, 132]
Puberty	During fetus development enormous hormonal changes occur which are associated with sexual maturation	Nutritional balance is linked to hormonal balance and can be achieved through improved family food behaviors	Introduces long term immunity	[133, 134, 135, 136]
Adults	prone to viral infections due to the hormonal imbalance, and non-communicable disease	Vitamin D	Anxiety, and depression can be reduced	[137, 138, 139]
Old	Depletion of zinc status	Zn supplement can be taken into account to build immunity	Blocks the replication of SARS CoV-2	[140, 141]
	Lower oxygen level	Iron-rich food along with vitamin B6 rich food should be consumed in proper portion	Assists to level up oxygen level	[142]
	Lower level of Vitamin D	Omega-3 along with Protectin D1, vitamin D and calcium, vitamin E, magnesium, folate can be supplemented as they all are interlinked to each other and increase vitamin D status	Reduces mortality rate through mitigating age related complications	[143, 148, 149]
	Dietary Fibers	Soluble fibers found in oats, barley, peas, apples, citrus fruits, and potatoes as well, Chemically engineered sulfated glucans	Mitigate their constipation and shows strong antiviral activities	[144, 145, 146, 147].
	Vitamin deficiencies	Supplementation through Vitamin C, Vitamin E, megavitamin D3 dose therapy	Used to treat patients during SARS epidemic to stimulate immunity	[73, 141]
	Abnormal Vitamin E and Vitamin D status allow frequent infections	Vitamin D, Vitamin C, Zinc, and Echinacea in combination must be taken on a regular basis	Worked better in common cold	[150, 151]
	Others	Tannins 3 times daily as tea or from fruits	Safe and highly effective antiviral reagents	[152, 153, 177]

### 2.5 Challenges towards the nutritional interventions during COVID19 pandemic

During every pandemic, people all over the world witness economic, social and mental pressure from country level to individual level due to sharp decline in GDP growth rate including a drop in domestic economic activity, a decline in exports of clothing and a fall in remittances from Bangladeshi living in abroad. Those factors have huge impact on health sector and research as well. No valid medicine or vaccine has been discovered to treat SARS CoV-2 infected people yet. COVID-19 pandemic has disrupted the supply chains and instigated financial hardship on distinct logistics companies as well as transportation resulting in poor availability of good nutritional food. COVID-19 has brought out tremendous financial troubles, irrespective of income. People both in developed and developing countries used to eat unhygienic street food, junk food and processed food with high chemicals and preservatives. In contrast, the quarantine during the pandemic induces binge eating among the rich which results in weight gain. Prolonged stay at home reduces physical activity and exposure to sun leading to low vitamin D in the body, one of the most attention seeker nutrient during this pandemic. In that case, modification of diet is the best approach to tackle this pandemic. However, to counteract the negative impact of reduced physical activities, people should refrain themselves from multiple meals a day with a long overnight fast. Avoiding refined sugar and balanced consumption of protein, seeds and vegetables will be helpful to modulate the immune function to fight against inflammation. Older people need to take more protein than the young ones and it is recommended to take at least 1.0 g/kg body weight to maintain muscle mass which may increase in presence of chronic illness. Supplementation strategy with vitamins and minerals should be implemented to overcome the malnutrition of aged ones due to inability of ingesting adequate energy with food. But the saddest part is, without income, it is almost impossible to ensure proper nutrition and healthy lifestyle. Several countries around the world used certain nutritional supplementations in clinical settings to assess their true impact on COVID-19 patients. Unfortunately, all of those trials were predominantly carried out in hospital setting with small sample size which followed a cross-sectional prospective design. The first step to battle against COVID-19 is to successfully identify the Corona positive cases. In least developed countries like Bangladesh, even the medical technologists are not enough trained to collect sample in a right way to symbolize the true positive Coronavirus cases, which is one of the biggest challenges. Some essential nutrients for instance-vitamin A, D, E, Zinc, vitamin B complexes were used against Avian Coronavirus, Bovine Coronavirus, SARS-CoV and MERS [176, 178], which were mostly responsible for the epidemic occurrences in the past few decades. During the COVID-19 pandemic, a very few of them have been trialed clinically on hospitalized patients, but their biggest limitations were their small cohort and lack of randomized control trials due to lack of funding, time consumption and expensiveness. Albeit, all of those nutrient supplement trials should be conducted in a large scale to bring out the true exposure and outcome effects. Apart from those, one of the major challenges is the lack of public awareness towards taking proper food in a proper quantity and discussing it with dietitians. Especially, in the least developed country like Bangladesh, people mostly depend on the doctors only when there arises any physical complications and food intake related discussions. In a word, the main gaps are mostly correlated to lack of diversified research designs with a priority on both laboratorial and hospital-based studies. In a densely populated as well as developing country like Bangladesh, it is more challenging to improve health sector overnight and increase Corona tests and isolate the positive cases as there is always an economic pressure on the underdeveloped and least developed countries.

## 3 Conclusion

Nutritional interventions play a central role in boosting up immunity and preventing infections among all aged groups. In many cases, a single nutrient deficiency can be connected with compromised immunity and increased susceptibility to infections whereas multiple nutrients deficiency may lead to more complex and serious health complications in human body. Dietary modifications such as reduced carbohydrate intake and consuming a small amount of fat in diet than the recommended dietary intake may promote immune system resulting in reduction frequency and severity of infectious diseases. Therefore, dietary strategies can serve as a therapeutic tool to reduce the morbidity and mortality rate caused by COVID-19. Here, we have accumulated the proof of different dietary strategies to combat not only this pandemic but also post pandemic situation. In this work, we have found a great consensus that both individual nutrients as well as a combination of multiple nutrients can be supplemented to modulate the severity of COVID-19 at individual level. Furthermore, a community level as well as country level dietary guidelines for at risk populations may help to modulate the trajectory of COVID-19 pandemic both at national and global level. This current work can be used as a resource for different nutrients and their functions, dietary sources (plant, animal or others) and recommended intake for different age groups. Since we get most nutrients from natural sources, we should conserve natural sources to be alive.

## Declarations

### Author contribution statement

All authors listed have significantly contributed to the development and the writing of this article.

### Funding statement

This research did not receive any specific grant from funding agencies in the public, commercial, or not-for-profit sectors.

### Data availability statement

Data included in article/supplementary material/referenced in article.

### Declaration of interests statement

The authors declare no conflict of interest.

### Additional information

Mahmudul Hasan, one of the corresponding authors of this article is expressing his utmost gratitude and love to his wife Momotaj Begum Jui. They got married on December 4, 2020, and Hasan had to submit the final revision of this article on December 8, 2021, which was the deadline from Heliyon and the following date of their wedding reception.
